# Supplementary figures and images for: Evolution of corneal transplantation techniques and their indications in a French corneal transplant unit in 2000–2020
Source: PLoS One. 2022 Apr 29;17(4):e0263686. doi: 10.1371/journal.pone.0263686 (PMC9053824; doi:10.1371/journal.pone.0263686)

**Supplementary Figure S1** Change in Patient Sex Over Time in the Whole Cohort.

**
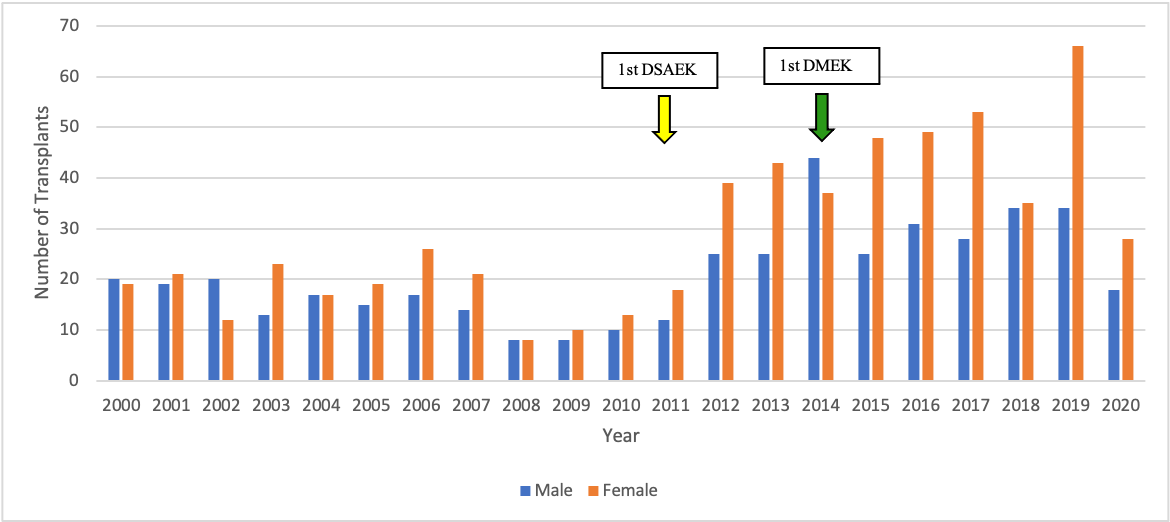
**

Supplement: S1 Fig — (DOCX) [file pone.0263686.s001.docx]

**Supplementary Figure S2** Change in Patient Sex Over Time in PKP

**
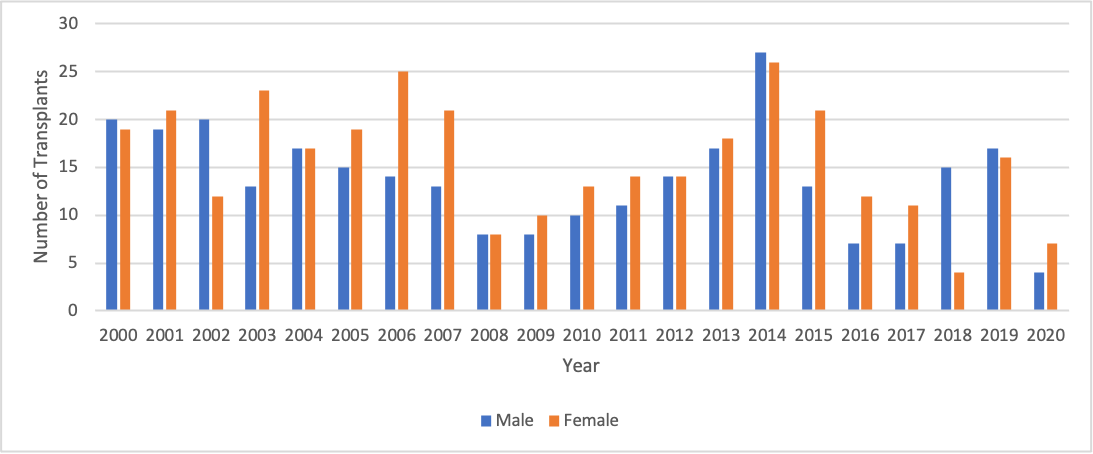
**

Supplement: S2 Fig — (DOCX) [file pone.0263686.s002.docx]

**Supplementary Figure S3** Change in Patient Sex Over Time in DSAEK

**
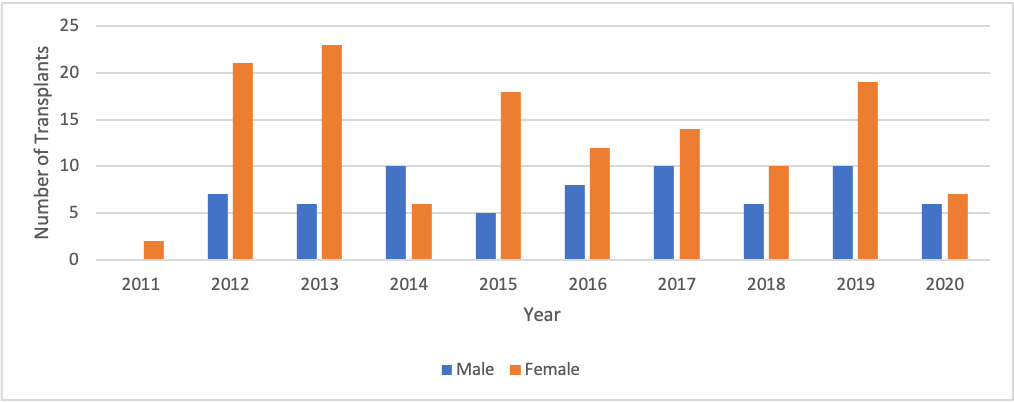
**

Supplement: S3 Fig — (DOCX) [file pone.0263686.s003.docx]

**Supplementary Figure S4** Change in Patient Sex Over Time in DMEK.

**
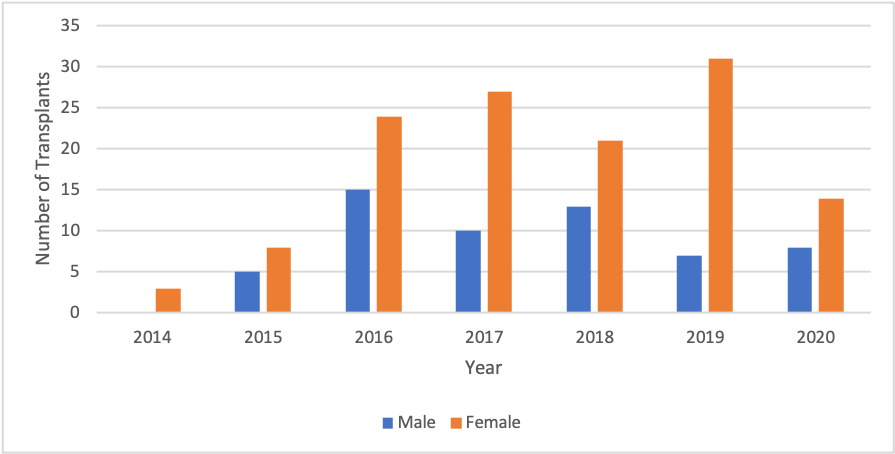
**

Supplement: S4 Fig — (DOCX) [file pone.0263686.s004.docx]

**Supplementary Figure S5** Change in Patient Age Distribution Between 2000-2010 and 2011-2021

**
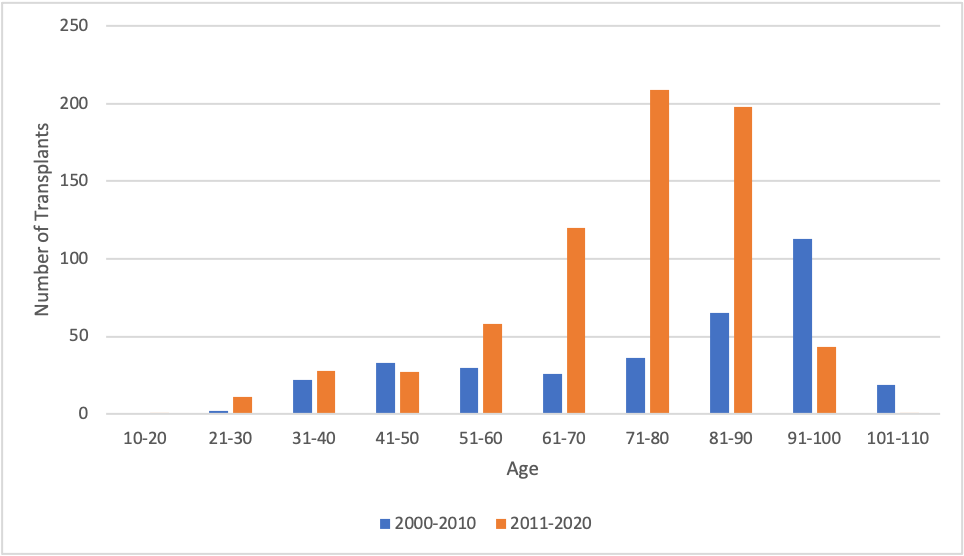
**

Supplement: S5 Fig — (DOCX) [file pone.0263686.s005.docx]
