# Supplementary material for: Evolution of corneal transplantation techniques and their indications in a French corneal transplant unit in 2000–2020
Source: PLoS One. 2022 Apr 29;17(4):e0263686. doi: 10.1371/journal.pone.0263686 (PMC9053824; doi:10.1371/journal.pone.0263686)
Supplement: S1 Table — (DOCX) [file pone.0263686.s006.docx]

**Supplementary Table S1.** Summary of the literature studying the evolution of keratoplasty techniques and indications before and after DSAEK and DMEK were introduced.

| **Author year country [ref]** | **No. of patients, source** | **Study period** | **Mean/median patient age, years**  **% Female sex** | **Most common indications at start (% of total for indicated period)** | **Most common indications at end (% of total for indicated period)** | **Trends in surgical techniques** | **Rise in annual kp cases, fold change** |
| --- | --- | --- | --- | --- | --- | --- | --- |
| Boimer 2011  Canada^20^ | 7755  Eye Bank of Canada | 2000-2009 | 69  52% | 2000-2001  1. BK (31%)  2. Regraft (18%)  3. FECD (13%)  4. KC (12%) | 2008-2009  1. Regraft (26%)  2. BK (24%)  3. FECD (22%)  4. KC (11%) | -DSAEK introduced 2004-2005  -DSAEK rose to 35%, for BK (to 50% of cases), FECD (to 67%) & regrafts (to 22%)  -DALK introduced 2006, rose to 3%  🡪 PKP dropped (from 100% to 64%)  -DMEK not yet introduced | 1.1-fold increase in 2000-2001 vs 2008-2009 |
| Keenan 2011 UK^23^ | NA  NHS Blood and Transplant | 1999-2009 | NA (bimodal distribution at 20-40 and60-80, especially for men)  NA (men predominate in most age categories for both PKP and EK) | NA | NA | -EK introduced 2003/2004, rose to 20%  -DALK rose slightly (3% to 14%)  🡪 PKP dropped (91% to 60%) | 1.2-fold increase in 2008/2009 vs 1999/200æ0 |
| Cunningham 2012 New Zealand^24^ | 2,205  New Zealand National Eye Bank | 2000-2009 | 45 (bimodal distribution at 20-29 and 70-79)  46% | 2000  1. KC (42%)  2. BK (17%)  3. Keratitis (12%)  4. Corneal dystrophy (9%)  5. Regraft (8%) | 2009  1. KC (41%)  2. Regraft (17%)  3. BK (14%)  4. Corneal dystrophy (11%)  5. Keratitis (8%) | -DALK increased (6% to 15%)  -DSAEK introduced in 2006, rose to 15%  🡪 PKP dropped (96% to 68%)  -DMEK not yet introduced | 1.4-fold increase in 2000-2009 vs 1990-1999 |
| Ting 2011 Scotland^25^ | 921  Ocular Pathology Lab. | 2001-2010 | 56  43% | 2001-2005  1. KC (25%)  2. Regraft (19%)  3. BK (14%)  4. Keratitis (13%)  5. FECD (12%) | 2006-2010  1. KC (31%)  2. Regraft (19%)  3. FECD (14%)  4. Keratitis (12%)  5. BK (10%) | -DALK increased (14% to 31%)  -DSAEK introduced in 2008, rose to 9%, rose for FECD (to 36%) & BK (to 21%)  🡪DSAEK and DALK rose for regraft (4% to 18%)  🡪 PK decreased (from 95% to 60%)  -DMEK not yet introduced | 1.6-fold increase in 2001-2005 vs 2006-2010 |
| Tan 2014  Canada^21^ | 4,843  Eye Bank of British Columbia | 2002-2011 | 63  47% | 2002-2006  1. BK (20%)  2. Regraft (18%)  3. FECD (15%)  4. KC (15%) | 2007-2011  1. FECD (23%)  2. KC (17%)  3. Regraft (17%)  4. BK (15%) | -DSAEK introduced in 2007, rose to 39%, for FECD (to 87%), BK (to 42%) & regraft (to 28%)  -very few DALK, little change  🡪 PKP decreased (100% to 61%)  -very few DALK, little change  -KC mainly treated with PK (3% with DALK)  -DMEK not yet introduced | 1.1-fold  increase in 2002-2006 vs 2007-2011 |
| Robert 2015 Canada^26^ | 3,459  Québec Eye Bank | 2000-2011 | 64  53% | 2000-2008  1. BK (28%)  2. FECD (21%)  3. KC (14%)  4. Viral keratitis (8%) | 2009-2011  1. FECD (36%)  2. BK (22%)  3. KC (11%)  4. Viral keratitis (8%) | -DSAEK introduced in 2007, rose to 48%, mainly for FECD (to 90%) and BK (50%)  🡪 PKP dropped (97% to 68%)  -KC mainly treated with PK (2% with DALK)  -DMEK not yet introduced | 2.6-fold increase in 2011 vs 2000 |
| De Sanctis 2016  Italy^27^ | 3,426  Eye Bank of Piedmont | 2002-2011 | 67  58% | 2002-2006  1. BK (17%)  2. Regraft (16%)  3. FECD (13%)  4. KC (36%) | 2007-2011  1. BK (21%)↑  2. Regraft (19%)↑  3. FECD (17%)↑  4. KC (27%)↓ | -EK (98% DSAEK) introduced in 2006, rose to 30% (for BK 0.8% to 62%, for FECD 2.2% to 68% & regraft 0% to 30%)  -only 14 cases of DMEK, recently introduced?  -DALK introduced in 2004, rose to 12%  🡪 PKP dropped (from 100% to 57%) | 1.3-fold increase in 2007-2011 vs 2002-2006 |
| Tan 2015 Singapor^28^ | 1,242 first grafts  Singapore Eye Bank | 1991-2012 | 54 (no difference in mean age for PKP, DALK, or DSAEK)  46% | 2000-2011  1. BK  2. FECD  3. KC | | -DSAEK introduced in 2006, rose to 44%  -DMEK introduced in 2012  -DALK introduced in 1991, rose to 27%  🡪 PKP dropped from 96% to 28% | 1.6-fold increase in 2005 vs 2012 |
| Dickman 2016 Netherlands^29^ | 5,115 BK & FECD cases  Netherlands Organ Transplantation Registry | 1998-2014 | 71-72  57-61% for BK, 58–63% for FECD | 1998-2006  1. BK (98%)  2. FECD (2%) | 2007-2014  1. FECD (83%)  2. BK (17%) | -EK introduced in 2006, rose to 98% for FECD and 70% for BK  🡪 PKP dropped to 2% for FECD and 30% for BK | 3-fold increase in 2013 vs 1998 |
| Röck 2017 Germany^30^ | 1,259  University Eye Hospital Tübingen | 2005-2015 | NA  NA | 2005  1. KC (49%)  2. FECD (8%)  3. BK (4%) | 2016  1. FECD (70%)  2. BK (9%)  3. KC (7%) | -DSAEK introduced in 2008  -DMEK introduced in 2009, immediately replaced DSAEK, overtook PKP in 2013, rose to 60%  🡪 PKP dropped (100% to 40%)  -DALK performed at very low levels | 2-fold increase from 2005 to 2016 |
| Kim 2017 New Zealand^54^ | 5,574  New Zealand National Eye Bank | 1991-2015 | NA (bimodal distribution but first peak 20-29 dropped and second peak 70-79 rose)  NA | 1991-1995  1. KC (45%)  2. BK (19%)  3. Keratitis (14%)  4. Trauma (8%)  5. Regraft (5%)  6. FECD (3%) | 2011-2015  1. KC (35%)  2. Regraft (23%)  3. FECD (17%)  4. BK (11%)  5. Keratitis (4%)  6. Trauma (3%) | -DSAEK introduced in 2007, rose to 31%  -DMEK introduced in 2015  -DALK introduced in 2006, rose to 7%  🡪 PKP dropped (100% to 62%) | 1.7-fold from 2005 to 2015 |
| Droutsas 2018 Greece^31^ | 1,382  Academic tertiary referral center | 1999-2015 | 62 (increasing over time)  Male predominance switched to female predominance in 2009 | 1999  1. BK (28%)  2. Scar (21%)  3. Regraft (20%)  4. KC (15%)  5. FECD (1%) | 2015  1. BK (46%)  2. Regraft (19%)  3. FECD (16%)  4. KC (5%)  5. Scar (2%) | -DSAEK introduced in 2009, rose to 59% at end  -DMEK introduced in 2013, rose to 19% at end  -EK rose for BK (0% to 90%) and FECD (0% to 100%)  -DALK not mentioned  🡪 PKP dropped (100% to 22%) | - |
| Bigan 2018 France^44^ | 46,658  French Biomedicine Agency database | 2004-2015 | 59  39% (males predominated in every year) | 2004  1. BK (28%)  2. KC (24%)  3. FECD (11%)  4. Regraft (9%) | 2015  1. FECD (23%)  2. BK (22%)  3. Regraft (17%)  4. KC (13%) | -EK introduced in 2004  -DALK rose for KC (7% to 54%)  -EK rose for FECD (1% to 70%), BK (0.4% to 53%), regraft (2% to 27%)  🡪 PKP dropped (95% to 50%) | 1.1-fold increase in 2014 vs 2004 |
| Flockerzi 2018  Germany^32^ | 78,165  German Ophthalmology Society Registry | 2001-2016 | NA  NA | NA | NA | -EK introduced in 2006, rose to 57%  -DSAEK differentiated from DMEK in 2009, DMEK overtook DSAEK in 2012 (53% DMEK, 4% DSAEK in 2016)  🡪 PKP dropped (96% to 40%)  -DALK rose from 3% to 6% in 2011, then fell to 3% | 1.5-fold increase in 2016 vs 2001 |
| Australian Corneal Graft Registry Report 2018^33^ | 33,920  Australian Corneal Graft Registry | 1997-2017 | NA (unimodal increase in age, peak at 70-79)  26% | NA | NA | -DSAEK introduced in 2006, rose to 34%  -DMEK introduced in 2008, rose to 19%  -DALK introduced in 2001, rose to 10%  🡪 PKP dropped (94% to 35%) | 1.5-fold from 1997-2006 to 2007-2017 |
| Javadi 2020 Iran^34^ | 115,743 Central Eye Bank of Iran | 1991-2017 | NA  39% | 1. KC (40%)  2. BK (19%)  3. Corneal scar & opacities (16%)  4. Regraft (8%)  5. Corneal dystrophies (5%; FECD, 1%)  -Sign. increase in KC, BK, regraft  -Sign. decrease in corneal scar & opacities, corneal dystrophies | | -DSAEK introduced in 2007, rose to 24%  -DMEK introduced in 2013, rose slightly to 1%  -DALK dropped (from 17% in 2006 to 1%)  -no change in PKP (approx. 80%) | 1.5-fold increase from 2004-2006 to 2007–2017 |
| Park 2015 USA^55^  Eye Bank Association of America Report 2019^2^ | 459,528  Eye Bank Association of America (2014) | 2005-2014 (2019) | NA (unimodal increase in age, peak at 71-80)  40% | 2005  1. BK (14%)  2. KC (11%)  3. FECD (10%)  4. Regraft (9%) | 2019  1. FECD (36%)  2. Regraft (15%)  3. BK (10%)  3. KC (6%) | -DSAEK introduced in 2005, rose to 20% in 2019  -DMEK introduced in 2011, rose to 15% in 2019  -EK used for 96% FECD  🡪 PKP dropped (95% to 42% in 2019)  -KC mainly treated with PK (11% with DALK in 2019) | 1.7-fold increase in 2014 vs 2005  (1.9-fold increase in 2019 vs 2005) |
| Our study 2021  France | 1,042  Specialized Ophthalmology Department | 2000-2020 | 74 (U-curve, PKP, DSAEK, DMEK ages decreasing with time)  58% (females also largely predominate in PKP and especially DSAEK and DMEK) | 2000-2014  1. BK (38%)  2. Regraft (20%)  3. KC (17%)  4. FECD (10%) 5. Infection (10%)  6. Trauma (6%) | 2015-2020  1. FECD (40%)  2. BK (23%)  3. Regraft (23%)  4. Infection (6%)  5. Trauma (4%)  6. KC (3%) | -DSAEK introduced in 2011, rose to 27% in 2016-2020  -DMEK introduced in 2014, rose to 38% in 2019  🡪PKP dropped (100% to 17%)  🡪Vast majority of BK cases treated with DSAEK (100% PKP to 80% DSAEK)  🡪All FECD cases treated with DMEK (100% with PKP to 100% DMEK)  🡪Regraft sometimes treated with DSAEK (44%) or DMEK (24%) | 2.2-fold increase from 2000-2010 to 2011-2020 |
