# Supplementary material for: Evolution of corneal transplantation techniques and their indications in a French corneal transplant unit in 2000–2020
Source: PLoS One. 2022 Apr 29;17(4):e0263686. doi: 10.1371/journal.pone.0263686 (PMC9053824; doi:10.1371/journal.pone.0263686)
Supplement: S2 Table — (DOCX) [file pone.0263686.s007.docx]

**Supplementary Table S2.** International changes in PKP, DSAEK, and DMEK use and indications.

| **Country (ref) N** | **Increase in cases** | **Year DSAEK introduced** | **Year DMEK introduced** | **Year DALK introduced** | **Study period (total years)** | **Change in PKP from first to last timepoint** | **Increase in DSAEK at last timepoint** | **Increase in DMEK at last timepoint** | **Change in DALK from first to last time point** | **Use of DSAEK for BK at end** | **Use of DSAEK for FECD at end** | **Use of DSAEK for regraft at end** | **Use of DMEK for FECD at end** | **Change in indication** |
| --- | --- | --- | --- | --- | --- | --- | --- | --- | --- | --- | --- | --- | --- | --- |
| Canada^20^ | 1.1 | 2004-5 | - | 2006 | 2000-2009 (10) | 100🡪64% | 35% | - | 0🡪3% | 50% | 67% | 22% | - | BK-regraft-FECD  Regraft-BK-FECD |
| UK^23^ | 1.2 | 2003-4 | ? | Before 1999 | 1999-2009 (11) | 91🡪60% | 20% | - | 3🡪14% | - | - | - | - | - |
| Scotland^25^ | 1.6 | 2008 | - | Before 2000 | 2000-2010 (11) | 95🡪60% | 9% | - | 6🡪15% | 21% | 36% |  | - | KC-regr-BK-kerat-FECD  KC-regr-FECD-kerat-BK |
| Canada^21^ | 1.1 | 2007 | - | Before 2002 | 2002-2011 (10) | 100🡪61% | 39% | - | Very few | 42% | 87% | 28% | - | BK-regr-FECD-KC  FECD-KC-regr-BK |
| Canada^26^ | 2.6 | 2007 | - | ? | 2000-2011 (12) | 97🡪68% | 48% | - | Very few | 50% | 90% | - | - | BK-FECD-KC  FECD-BK-KC |
| Singapore^28^ | 1.6 | 2006 | 2012 | 1991 | 1991-2012 (22) | 96🡪28% | 44% | Just intro. | 0🡪44% | - | - | - | - | - |
| Netherlands^29^ | 3.0 | 2006 | ? | - | 1998-2014 (17) | - | - | - | - | EK 70% | - | - | EK 98% | BK-FECD  FECD-BK |
| Germany ^30^ | 2.0 | 2008 | 2009 | ? | 2005-2015 (11) | 100🡪40% | DSAEK replaced with DMEK immed | 60% | Very few | - | - | - | - | KC-FECD-BK  FECD-BK-KC |
| New Zealand^54^ | 1.7 | 2007 | 2015 | 2006 | 1991-2015 (25) | 100🡪62% | 31% | Just intro. | 0🡪7% | - | - | - | - | KC-BK-kerat-trau-reg-FE  KC-reg-FE-BK-kerat-trau |
| Greece^31^ | - | 2009 | 2013 | - | 1999-2015 (17) | 100🡪22% | 59% | 19% | - | EK 90% | - | - | EK 100% | BK-scar-reg-KC-FECD  BK-regr-FECD-KC-scar |
| France^44^ | 1.1 | 2004 EK | ? | Before 2004 | 2004-2015 (12) | 95🡪50% | 57% | - | ? | EK 53% |  | - | EK 70% | BK-KC-FECD-regr  FECD-BK-regr-KC |
| Germany ^32^ | 1.5 | 2006 | ? | Before 2001 | 2001-2016 (16) | 96🡪40% | 4% | 53% | 3-6-3% | - | - | - | - | - |
| Australia ^33^ | 1.5 | 2006 | 2008 | 2001 | 1997-2017 (21) | 94🡪35% | 34% | 19% | 10% | - | - | - | - | - |
| Iran^34^ | 1.5 | 2007 | 2013 | Before 2006 | 1991-2017 (27) | No change 40% | 24% | 1% | 17🡪1% | - | - | - | - | - |
| USA^2^ | 1.7 2014  1.9 2019 | 2005 | 2011 | - | 2005-2019 (15) | 95🡪42% 2019 | 20% | 15% | Very few | - | - | - | EK 96% | BK-KC-FECD-regr  FECD-regr-BK-KC |
| **Average international values** | **1.6 (1.1–3)** | **2006 (2004-2009)** | **2011 (2008-2015)** |  |  | **51% at end (22–68%)** | **31% (4–59%)** | **28% (1–60%)** |  | **63% (21–90%)** | **70% (63–90%)** | **27% (22–30%)** | **91% EK (70–100%)** |  |
| Present study, France | 2.2 | 2011 | 2013 | Before 2000 | 2000-2020 (21) | 100🡪27% | 19% | 46% | Very few | 80% | 0% | 44% | 100% |  |
